# Epiphytic Yeasts from South Romania for Preventing Food Microbial Contamination

**DOI:** 10.3390/life14091087

**Published:** 2024-08-29

**Authors:** Viorica Maria Corbu, Andreea Ștefania Dumbravă, Irina Gheorghe-Barbu, Ortansa Csutak

**Affiliations:** 1 Department of Genetics, Faculty of Biology, University of Bucharest, Aleea Portocalelor 1-3, 060101 Bucharest, Romania; viorica-maria.corbu@bio.unibuc.ro; 2 Research Institute of University of Bucharest (ICUB), University of Bucharest, B.P. Hasdeu Street 7, 050568 Bucharest, Romania; irina.gheorghe@bio.unibuc.ro; 3 Department of Technological Irradiation (IRASM), Horia Hulubei National Institute of Physics and Nuclear Engineering—IFIN-HH, 077125 Măgurele, Romania; andreeadum29@gmail.com; 4 Department of Microbiology and Botany, Faculty of Biology, University of Bucharest, Aleea Portocalelor, no. 1–3, 060101 Bucharest, Romania

**Keywords:** stress resistance, genetic dimorphism, yeasts pathogenicity, antibacterial activity, phytopathogenic filamentous fungi, crop contamination, food borne pathogens

## Abstract

Epiphytic yeasts represent an important source for the development of novel strategies aiming to combat food microbial contamination. The present study deals with the characterization of nine yeast strains belonging to *Starmerella*, *Candida*, *Metschinikowia*, *Lachancea*, *Kodamaea* and *Pichia* genera, isolated from the surface of plants from the Botanical Garden “Dimitrie Brandza” (Bucharest, Romania) for use as antimicrobial and probiotic agents. The tests involved the determination of the safe status, cell growth under stress conditions, and activity against pathogenic *Candida* and bacteria strains, respectively, as well as phytopathogenic filamentous fungi and lipolytic activity. None of the nine strains showed all the characteristics for virulence and pathogenicity, with the rare positive results being explained rather by their adaptability to the habitats of origin. The strains *Lachancea thermotolerans* CMGB-ST12 and *Kodamaea ohmeri* CMGB-ST19 grew at 37 °C; *Metschnikowia reukaufii* CMGB-ST21.2, *M. reukaufii* CMGB-ST.8.1 and *M. reukaufii* CMGB ST10 grew in the presence of 10% NaCl, while *L. thermotolerans* CMGB-ST12 and *K. ohmeri* CMGB-ST19 tolerated both acidic and alkaline pH values well (3.0 to 12.0). The studied yeast strains showed good antimicrobial activity against *Candida krusei*, *Candida albicans* and Gram-negative bacterial strains, with *K. ohmeri* CMGB-ST19 and *Pichia membranaefaciens* CMGB-ST53 inhibiting up to 100% the development of filamentous fungi. All the strains produced lipases for tributyrin hydrolysis, the best producer being *Starmerella bombi* CMGB-ST1, and only *Candida magnoliae* CMGB-ST8.2 tested positive against other probiotic yeasts. Overall, our nine yeast strains show high potential for industrial applications, for obtaining probiotic products and for preventing the development of a wide range of microbial food contaminants.

## 1. Introduction

Microbial resources are an advantage for modern society that needs to be utilized from both a commercial and ecological standpoint. Due to their widespread distribution, the microorganisms present a high capacity for improving numerous biotechnology-based industries or research domains such as food production, biomedicine, bioremediation, biochemistry or agriculture [1]. Among the biotechnologically relevant microorganisms, yeasts are of great interest, due to their limited pathogenic potential. A significant number of yeasts with biotechnological potential have been recovered from phyllosphere, which includes all the aboveground organs of a plant, such as buds, leaves, flowers, fruits or seed [2]. The research directions in the study of microorganisms, regarding the phytobiome of plants across different geographical regions, have primarily concentrated on filamentous fungal and bacterial communities; nevertheless, a multitude of yeast species have been demonstrated to play a significant role in these communities. In order to survive in hostile environments, the epiphytic yeasts typically utilize resources provided from plants, such as carbohydrates, amino acids, organic acids, and salts [3]. The yeasts can produce a wide range of hormones (auxins, gibberellins and cytokinins) or other chemical compounds (enzymes such as cutinases, esterases, lipasese or iron-chelating siderophores) that might lead the plant cell wall to become unstable, allowing the food supply to become accessible [4]. Both the age of the plant and the species to which it belongs have a significant impact on the microbial biodiversity on the surface of the vegetative organs of plants [5]. Strong natural selection effects are also attributed to abiotic causes. Microorganisms compete with one another for substrate due to exposure to various radiation types, levels of pollution in the surrounding environment, and nitrogen fertilizers [6]. The isolation of new strains with special metabolic capacities induced by exposure to different stressors can represent an invaluable alternative, not only for the development of biocontrol products but also for the optimization of biotechnological processes in the food industry and beyond.

Among the most famous genera of yeasts frequently isolated from the surface of plants, the genus *Metschnikowia* stands out. The strains belonging to this genus, more precisely to the species *M. pulcherrima*, have proven extremely useful as biocontrol agents, being able to inhibit the growth of fungi such as *Pestalotiopsis vismiae*, *Botrytis cinerea*, *Alternaria alternata* or *Penicillium expansum* [7,8]. *M. reukaufii* is another *Metschinikowia* species associated with flowers and floral nectars [9,10], and is currently being investigated for the production of microbial enzymes for the food and chemical industries, such as α-galactosidases [11] or other mixtures of enzymes used for obtaining bioactive oligosaccharides with prebiotic functions [12]. *Pichia membranaefaciens* is another yeast with high potential for the protection of plants, although it is more frequently isolated from fermented foods (such as the traditional Nigerian foods burukutu, ogi or pito [13]; fermented pickles [14]; fermented fish, shrimp, meat or cereals [15]) or industrial wastewaters and soil [16]. Its importance was proven for the research direction of biocontrol, described as being able to inhibit the growth of *Penicillium expansum*, *Penicillium digitatum*, *Botrytis cinerea*, *Rhizopus stolonifer*, and *Monilinia fructicola* [17,18,19]. Other yeasts associated with plants are *Debaryomyces hansenii* and *Wicherhamomyces anomalus*. The members of these species show high resistance to stress factors such as pH variations, high salt concentrations, and high temperature variations. Both species have been characterized as producing killer toxins that can inhibit the development of fungi such as *Monilinia fructicola* that affect the post-harvest quality of apples, peaches and plums. These yeasts intervene in the primary mechanism of plant protection against phytopathogens by stimulating the activity of peroxidases that protect plant tissues against reactive oxygen species, produced as a result of phytopathogen action [20]. Moreover, it has been proven that yeasts isolated from the surface of plants can be successfully used as an inhibitor of mycotoxin production by mycotoxigenic fungi, thus preventing the accumulation of these particularly dangerous compounds for consumers in fruits [21]. A study carried out by Islam et al. [22] revealed that epiphytic and endophytic yeasts such as *Pichia fermentas* and *Meyerozyma caribbica*, isolated from the surface of pineapple fruits and from its pulp, can also contribute to the improvement of biochemical and hematological parameters in the case of fish species such as *Barnonymus gonionotus*, thus having a positive economic impact in terms of fish farming. Due to their high metabolism, the epiphytic yeasts can be used for the benefit of society, with an emphasis on developing innovative strategies to improve the quality of food. Whether we talk about their role in fermentation processes, the production of secondary metabolites with applications in developing food product varieties, or focus on their highly diversified antimicrobial activity, yeasts represent a potentially invaluable resource for the transition to a sustainable and innovative food industry. The benefits of yeasts in food production are multifaceted. On the one hand, yeasts are essential in the production of fermented beverages or foods, and on the other hand, they can contribute to the nutritional value of food by synthesizing vitamins and enhancing the bioavailability of minerals. They also produce enzymes and functional metabolites that can improve food quality and extend shelf life [23,24]. Furthermore, the yeasts represent a good alternative for the commercially available probiotic products, containing almost exclusively lactic acid bacterial strains. The major advantage of probiotic yeasts is the fact that they are not affected by antibiotics and, therefore, the propagation of antibiotic-associated human intestinal disease and the antibiotic resistance can be reduced [25].

One of the current research directions in the protection of food against microbial contamination aims to develop alternative strategies based on the exploitation of the natural antagonism between microorganisms, so as to avoid the use of synthetic compounds. Yeasts’ microbial group might represent an important reservoir of new strains with important and versatile antimicrobial properties, and the continuous search for new species/strains with enhanced potential is considered essential at present, due to the fact that it facilitates both the improvement of antimicrobial strategies by expanding the activity spectrum and the preservation of microbial biodiversity by limiting the extensive use of chemical synthesis compounds. In this context, the present study focuses on the characterization of epiphytic yeasts as alternatives for developing new strategies to prevent food microbial contamination. Nine epiphytic yeast strains, isolated from South Romania and included in the collection of microorganisms of the Department of Genetics, Faculty of Biology, University of Bucharest, Romania, were tested to determine their growth under stress conditions, evaluating their pathogenicity potential and characterizing their antimicrobial activity against human pathogenic and potential pathogenic *Candida* and bacterial strains, and against phytopathogenic filamentous fungi. 

## 2. Materials and Methods

### 2.1. Biological Material

Nine yeast strains previously identified using conventional taxonomy tests (Table S1), which were previously isolated from the phyllosphere of plants taken from the Bucharest “Dimitrie Brândză” Botanical Garden, were selected from the Collection of the Research, Training and Consultancy Group in Microbiology, Genetics and Biotechnology, Department of Genetics, University of Bucharest (CMGB), and included in the present study. The yeasts were stored at −70 °C in a Revco LegaciTM Refrigeration System (ThermoFisher Scientific, Copeland, UK) on yeast peptone glucose (YPG) medium with 20% glycerol. Prior to use, the yeast strains were cultivated on YPGA slants YPG culture media with 1.5% agar (BD Difco™; Becton, Dickinson and Company; Franklin Lakes, NJ, USA) for 24 h at 28 °C.

### 2.2. Taxonomic Confirmation of Yeast Strains Using Automated Systems and Molecular Analyses

#### 2.2.1. Taxonomic Identification Using the Biolog YT MicroPlates System (Biolog Inc., Hayward, CA, USA)

The Biolog system was used in order to generate dendrograms focused on the phenotypic phylogeny, resulting from the assimilation and fermentation tests of the carbon sources arranged in the wells of the YT Biolog plates (Biolog, Inc., Hayward, CA, USA). The inoculum and assays were processed using the protocol described by the manufacturer. From the 24 h cultures (YPGA medium; 28 °C), an inoculum with 47 transmittance was prepared in sterile distilled water. Then, 100 µL suspensions were transferred to the wells of the YT Biolog plate and then incubated at 28 °C for 3 days. The results were monitored daily for 72 h using MicroStation™ (Biolog, Inc., Hayward, CA, USA), and the data analysis was performed using Microlog Software version 4.2 (Biolog, Inc., Hayward, CA, USA). For the validation of the results and the working method, the reference strain *C. albicans* ATCC10231 was used as control. 

#### 2.2.2. Taxonomic Identification Using the Bruker MALDI-TOF (Matrix-Assisted Laser Desorption/Ionization–Time of Flight) Identification System (Bruker Corporation, 40 Manning Road, Billerica, MA, USA)

The MALDI-TOF MS identification technology identifies a species-specific protein profile, which is then compared to a database including results from reference strains [26]. Fresh cultures of yeasts (YPGA complex growth medium at 28 °C; 24 h) were processed for analysis following the manufacturer’s instructions. The protein profile was determined using the Microflex III system (Bruker Daltonik, Bremen, Germany), and the results were compared to those in the MALDI BioTyper Software database (MALDI Biotyper^®^ Reference Library version 2023) (Bruker Daltonics, Billerica, MA, USA). The results were expressed as scores ranging from 0 to 3, considering only scores above 2 as meaningful for species identification [27]. 

#### 2.2.3. Molecular Taxonomy Identification Based on ITS1-5.8S rDNA-ITS2 Coding Region

Genomic DNA was isolated using the LiCl method described by Csutak and Vassu, [28]. The DNA samples concentrations were determined at λ 260 nm using a NanoVue Plus spectrophotometer (GE HealthCare Technologies, Inc., Chicago, IL, USA). For the PCR-RFLP analysis and sequencing technique, the ITS1-5.8S rDNA-ITS2 coding region was amplified using the ITS1 (5′-TCCGTAGGTGAACCTGCGG) and ITS4 (5′-TCCTCCGCTTATTGATATGC) (200 pM, TIB MOLBIOL) primers. The amplicons were then digested with four restriction endonucleases: *Cfo*I (5′-GCG/C-3′), *Hinf*I (5′-G/ANTC-3′), *Hae*III (5′-GG/CC-3′), and *Msp*I (5′-C/CGG-3′) (10 U/µL, Promega, Madison, WI, USA). The restriction fragments were separated by gel electrophoresis (1.7% agarose and 0.5× Tris-Boric acid-EDTA (TBE) as buffer) and then analyzed using PyElph software v. 1.4 [29]. To corroborate the results of the automatized identification systems and the PCR-RFLP approach, the ITS1-5.8S rDNA-ITS2 amplicons of the ST1, ST21.2, ST19, ST12, ST8.2 and ST53 strains were also sequenced. Sanger sequencing was performed by the CeMIA-Cellular and Molecular Immunological Applications Company (Larissa, Greece) and the contigs were assembled using Bioedit version 7.7.1 (Informer Technologies Inc., Altamor Drive, Los Angeles, CA, USA). Fasta sequences are available on the GenBank database (accession numbers: PQ202863; PQ202864; PQ202865; PQ202866; PQ202867). The sequences obtained were aligned with GenBank, using the BLASTn algorithm for establishing the genera and species to which the analyzed strain belongs [30].

### 2.3. Confirmation of Safe Status

For the evaluation of the pathogenic potential of the microbial strains, we determined their ability to secrete hydrolytic enzymes, to transition to the form of pseudohyphae/hyphae, respectively, and their adherence capacity to the inert substratum. 

#### 2.3.1. Production of Hydrolytic Enzymes

For testing the production of hydrolytic enzymes, a protocol adapted from Corbu and Csutak [31], Zajc et al. [32], and Avram et al. [33] was used. An inoculum with 1McFarland density was obtained, starting from a fresh 24 h culture on YPGA medium. The suspension (10 µL) was spotted on the surface of culturing media specific for determining the ability to secrete specific hydrolytic enzymes: *hemolysins* (on Sabouraud Dextrose Agar medium (SDA) (Oxoid) (Hampshire, UK) with 50 mL/L ram blood); *amylases* (on YP culture media containing 0.5% yeast extract (Carl Roth, Karlsruhe, Germany), 1% bacteriological peptone (Sigma Aldrich, Burlington, MA, USA) and 1% NaCl (Carl Roth) supplemented with 10% starch); *caseinases* (on YP medium with 10% casein from milk (Carl Roth)); *extracellular deoxyribonucleases* (*DN-ases*) (on DNase Test Agar medium—Difco, East Rutherford, NJ, USA, supplemented with 0.01% toluidine blue (Sigma Aldrich, Burlington, MA, USA)); *phospholipases* (on SDA culture media with 100 mL/vL egg yolk, 1.17 g/L NaCl (Carl Roth, Karlsruhe, Germany), and 0.001% CaCl_2_∙2H_2_O (Carl Roth)); *gelatinase-type proteolytic enzymes* (on a specific culturing media containing 0.1% yeast extract; 0.5% sodium taurocholate (Sigma Aldrich, Burlington, MA, USA); 1% bacteriological peptone; 0.1% NaCl; 3% gelatin (Sigma Aldrich) and 1.5% agar-agar). After inoculation, the plates were incubated at 37 °C for 72 h and monitored daily to observe a positive reaction according to the reference protocols (the appearance of a clear area around the culturing spot for the hemolysins production; the appearance of a clear zone after staining with Lugol solution (Carl Roth) for amylase production; the presence of clear halos or a white precipitate around the colonies for the production of caseinases, gelatinases and phospholipases; or a light halo/ pale pink halo for the DN-ase production test). The results were recorded as arbitrary units (“−” negative result; “+” positive result, registered after the estimated time, “D”—delayed positive results; the positive result was registered after the optimal time of determination) at the end of the incubation time. Since the yeast strains were isolated from the surface of different plants, the production of the siderophore-like compounds was also determined using a specific culturing media (1% bacteriological peptone, 0.1% ammonium ferric citrate (Sigma Aldrich, Burlington, MA, USA), 2% agar) supplemented with 0.1% esculin (Sigma Aldrich, Burlington, MA, USA). The positive result in this case is indicated by the appearance of a brown to black colored zone around the yeast spot after at least 24 h of incubation at 37 °C. 

#### 2.3.2. Cellular Dimorphism Assays

The morpho-genetic dimorphism was determined using RPMI 1640 medium (Roswell Park Memorial Institute) (Sigma Aldrich, Burlington, MA, USA) with L-glutamine, no sodium bicarbonate, and 100 mL/L fetal bovine serum (Sigma Aldrich, Burlington, MA, USA). For obtaining mid-exponential growth phase cells, the yeast strains were cultured on YPG 24 h at 37 °C at 150 rpm, and then the cultures were centrifuged at 7000 rpm for 5 min. The cellular pellet obtained was dissolved in RPMI 1640 media and diluted up to 5 × 10^6^ cells/mL. After 3 h of incubation (37 °C), the hyphal percentage was calculated by counting the cells in hyphal/pseudohyphal form in 10 randomly selected microscopic fields and comparing them to the total number of cells counted. The microscopic analysis was performed using an optical microscope equipped with a 40× objective (MICROS, Veit an der Glan, Austria) [33].

#### 2.3.3. Adherence Capacity to Inert Substratum

The ability of each yeast strain to adhere to polypropylene substrate was assessed using the protocol described by Jin et al. [34]. From fresh yeast cultures (18 h at 37 °C; 150 rpm) a total volume of 1.5 mL was centrifuged (3 min at 8000 rpm). The cellular pellet was washed twice with 0.85% NaCl solution and then suspended in Yeast Nitrogen Base culture media with amino acids (L-histidine; DL-methionine; DL-tryptophan) (YNB-Sigma Aldrich, St. Louis, MO, USA) (6.7 g/L with 50 mM glucose up to 1 × 10^5^ cells/mL density. A total volume of 150 µL from the cell suspension was transferred to a 96-well polypropylene plate, followed by incubation for 72 h at 37 °C without shaking. When the incubation period ended, the cultures were discarded, and the wells were washed with 0.85% NaCl (Carl Roth, Karlsruhe, Germany), solution (three times) for the complete removal of the non-adhered cells. The adhered cells were fixed with pure methanol for 10 to 15 min, and then stained with 1% crystal violet solution (150 µL) for 15 min. After the removal of the dye, each well was washed with sterile distilled water (four times) and the attached cells were suspended in of 33% acetic acid (150 µL). The absorbance at 490 nm was determined with the Synergy™ HTX Multi-Mode Microplate Reader (Santa Clara, CA, USA). In order to validate the results, the protocol was repeated three times using three replica.

### 2.4. Cell Growth under Stress Conditions

Biomass accumulation under osmotic, ionic and thermal stress was determined spectrophotometrically by monitoring the optical density at 600 nm. For the ionic stress conditions, the yeast strains were cultivated on YPG broth with different pH values ranging from 3 to 12. The pH values were adjusted using 1 N HCl (Carl Roth, Karlsruhe, Germany), for pH < 6 and with 12 M NaOH for the pH values > 7. For the induction of osmotic stress, YPG medium with 0.5% to 12% NaCl was used as culturing media. In both cases, the inoculum (1%) was represented by a cell suspension with OD_600nm_ equal to 1, and the final volume of cultivation was 300 µL. The positive control for the growth was obtained by culturing the yeast strains on unmodified YPG culture media (pH 6.2 and no NaCl). After inoculation, the plates were incubated at 28 °C for 24 h, and the growth was determined by measuring the absorbance at 600 nm with a Synergy HTX microplate reader. For the accumulation of biomass in presence of thermal stress, the protocol used was similar to those described above, with the alteration that in this case the culturing media was represented by unmodified YPG media, but after inoculation the plates were incubated at 15 °C; 22 °C; 28 °C; 37 °C; 42 °C and 60 °C. Since yeast strains are generally considered mesophilic, the biomass accumulated at 28 °C was considered the positive control for the thermal stress assay C [31,35]. All the experiments were performed in triplicate and the results are expressed as mean ± standard deviation. For the statistical analysis, GraphPad Prism v9 was used. 

### 2.5. Qualitative Screening of Antimicrobial Activity 

The yeast strains were tested for their ability to inhibit the growth of different foodborne pathogens; pathogens that affect human health. The antimicrobial activity was determined against *Candida* strains, bacterial strains, and against phytopathogenic filamentous fungi. 

#### 2.5.1. Anti-Candida Activity

The yeast strains were tested for their anti-*Candida* activity against reference *Candida* strains (*C. parapsilosis* CBS604; *C. albicans* ATCC 10231; *C. krusei* CMGB 94) and against pathogenic *Candida* strains (*C. parapsilosis* CMGB-Y3; *C. krusei* CMGB-Y8; *C. albicans* CMGB-Y12; *C. catenulata* CMGB-Y7) which were previously isolated, characterized, and included in the CMGB collection by other researchers [33]. 

##### Anti-Candida Activity Based on Competition for Nutritive Substrates

The nine studied yeasts were cultured on YPGA media for 24 h at 28 °C, whereas the potentially sensitive *Candida* strains were cultured on YPG liquid medium for the same period but at 37 °C with agitation (150 rpm). When the incubation time ended, the liquid cultures were centrifuged (7 min at 7000 rpm) and the cell pellet was resuspended in sterile distilled water till OD_600nm_ 1. A 2 mL volume of the suspension was used to flood Petri dishes containing Yeast Malt Agar (YMA) medium (0.3% yeast extract; 1% D-glucose; 0.5% bacteriological peptone; 0.3% malt extract (Carl Roth, Karlsruhe, Germany),) so that the surface of the medium was completely covered with microbial suspension The surplus liquid was removed and the plates were dried. Then, an isolated colony of the tested yeast cultures was inoculated as a spot on the surface of the YMA culture media. The plates were then incubated at 28 °C for 7 days before being evaluated at regular intervals. The positive outcome is shown by the formation of a halo around the tested strain spot, which corresponds to growth inhibition of the *Candida* strain [36]. 

##### Anti-Candida Activity at Low pH Values

A similar protocol as the one described above was used for determining the antimicrobial activity at low pH values, with the alteration that in this case the culture media used was Killer medium, containing phosphate citrate buffer 0.1 M, 2% D-glucose; 1% yeast extract; 1% bacteriological peptone; 2% agar-agar and 0.05% methylene blue (Carl Roth, Karlsruhe, Germany),; pH 4.2 [37], and the tested strains were added as suspension of 2 McFarland prepared in sterile distilled water, starting from 24 h old cultures obtained on YPGA medium. The suspension was spotted (10 µL) on the surface of K-medium-inoculated Petri dishes and then the plates were incubated at 28 °C for 7 days. A positive result is indicated by the presence of a bright halo around the tested yeast spot, showing the total inhibition or partial inhibition of the potentially susceptible strain growth. For both assays, the results were recorded as arbitrary units as follows: “−”, lack of halo or halo size < 2 mm; “+”, 2–3 mm halo with medium luminous intensity; “++”, halo of 2–3 mm with high luminous intensity; “+++”, 5–6 mm complete halo. 

#### 2.5.2. Anti-Bacterial Activity

The antibacterial activity of the yeast strains was determined against both Gram positive (*Staphylococcus aureus* ATCC 6538; *Listeria monocytogenes* CMGB333; *Bacillus cereus* CMGB 53-100) and Gram negative bacteria (*Escherichia coli* ATCC 25922; *Pseudomonas aeruginosa* ATCC 27853; *Salmonella typhimurium* ATCC 14028). Bacterial strains were cultivated on liquid Luria medium containing 10 g/L peptone, 5 g/L yeast extract and 10 g/L NaCl (after adjusting the pH to 7.0 with 5 N NaOH (Sigma Aldrich, Burlington, MA, USA), the culture media was sterilized by autoclaving for 15 min at 121 °C) and incubated for 20 h at 37 °C. From the developed culture, a subculture was prepared and incubated for 3–4 h at 37 °C to reach OD_600nm_ between 0.4–0.6. This subculture was used by evenly spreading it on agar medium. Several medium variants were used, so that the medium that allows for the growth of both types of microorganisms (both yeasts and bacteria) could be selected. The media used were: Nutrient Agar (Oxoid); BHI—Brain Heart Infusion (Sigma-Aldrich); Muller–Hinton (Sigma Aldrich, Burlington, MA, USA)and YGSA [38]. The yeast strains were cultivated on liquid YPG medium for 24 h (28 °C), and centrifuged for 7 min at 7000 rpm. The cell pellet was dissolved in sterile distilled water and adjusted to OD_600nm_ 2. This suspension was spotted on the surface of the culture medium. The plates were incubated at 37 °C, and the yeasts’ antibacterial activity was assessed 24, 48, and 72 h after seeding. The growth of the two types of microorganisms was considered while interpreting the data, allowing the ideal growing medium to be chosen for the yeasts and bacteria examined.

#### 2.5.3. Preliminary Assessment of the Probiotic Potential of Yeast Strains

##### Lipolytic Capacity Screening Assays on Tributyrin Medium

The qualitative screening of the ability of yeast strains to release the butyric acid was carried out by determining the ability to hydrolyze tributyrin. Thus, to obtain isolated yeast colonies, the strains were streaked on YPGA medium and incubated for 24 h at 28 °C. An isolated colony was picked up and seeded on Yeast Nitrogen Base-Tributyrin Agar (YPTA) medium (pH-6.8: 67 g/L Yeast Nitrogen Base powder with amino acids, 0.5% (NH_4_)_2_SO_4_, 0.5% tributyrin (Carl Roth, Karlsruhe, Germany), 0.0125% Tween 80 (Carl Roth, Karlsruhe, Germany), and 2% agar) as a spot. The inoculated plates were incubated for one week at 28 °C, and monitored daily. A positive result is shown by the presence of a clear halo around the culture spot, resulting from the hydrolysis of tributyrin in the culture medium. The semi-quantitative determination of the lipolytic activity involved dividing the size of the halo formed by the size of the culture spot. Values equal to 1 indicate the absence of lipolytic activity, while values above 1 are directly correlated with the lipolytic capacity of the tested strains. For determining the impact of the growth temperature on the lipolytic activity, a similar assay was performed, following the previously mentioned protocol, with the amendment that in this case the incubation temperature of the strains inoculated on YPTA medium was varied. Thus, 3 temperature values were chosen, namely: 20 °C—the optimal temperature for industrial processes, 28 °C—the optimal temperature for the growth of yeasts, considered as a positive control in this case, and 37 °C—the temperature of the human body, which can indicate the potential application of produced lipases in the biomedical field [37]. 

##### Antimicrobial Activity against Probiotic Microorganisms

A similar protocol to the one described for the anti-*Candida* activity at low pH values was used for determining the impact on other microorganisms with potential probiotic abilities. Thus, 3 yeast strains: *S. boulardii* CMGB-S; *Kluyveromyces lactis* CMGB 112; *Kluyveromyces marxianus* CMGB 159 and 2 lactic acid bacteria (LAB): *Streptococcus salivarius* subsp. *thermophilus* ATCC 19258; and *Lactobacillus acidophilus* ATCC 4356 were selected for this assay and were considered as potential sensitive microbial strains. The potential sensitive strains were inoculated on the appropriate culture media, namely K-medium for the yeasts and Man–Rogosa–Sharpe (MRS) (Sigma Aldrich, Burlington, MA, USA)culture media (pH 6.5; 0.1% peptone from casein; 0.5% meat extract; 0.5% yeast extract; 0.2% K_2_HPO_4_, 0.1% Tween 80; 0.2% ammonium ferric citrate; 0.5% sodium acetate; 0.01% MgSO_4_ and 0.05% MnSO_4_) (Carl Roth, Karlsruhe, Germany), for the LAB strains. 

#### 2.5.4. Anti-Filamentous Fungi Activity

##### Qualitative Screening Tests for Antifungal Activity

The yeasts were also tested for their ability to inhibit both the sporulation and mycelial growth of *Botrytis cinereea, Rhizoctonia solani*, *Aspergillus ochraceus*, *Aspergillus flavus* GIS9, *A. flavus* GIS2, *A. flavus* GE2 and *A. flavus* T11 fungi (from the collection of the Faculty of Biotechnology of the University of Agronomic Sciences and Veterinary Medicine, Bucharest), which was determined using a protocol adapted from Marie et al. [39]. The yeasts were cultured on YPGA media (24 h at room temperature) while the filamentous fungi strains were cultured on PDA (Potato Dextrose Agar) (Sigma Aldrich, Burlington, MA, USA) for at least 7 days at room temperature, or until the growth and sporulation was significant. For the *B. cinerea* and *R. solani* strains, the inoculum was obtained by removing a 1 cm^2^ medium fragment from the passage made previously and sterile transferred to the center of the Sabouraud medium poured in a Petri dish. For the fungi with significant sporulation within 7 days, a spore suspension made in 0.85% NaCl (Carl Roth, Karlsruhe, Germany), solution, with a density equivalent to 1 McFarland, was used as inoculum. From this suspension, 10 µL were taken and arranged as a spot in the center of the Petri dish with medium. Subsequently, yeast strains taken from YPGA medium were inoculated radially, starting from the fungal spot. The incubation of the plates was carried out at room temperature (approximately 22 °C) and the monitoring of the antifungal action was carried out periodically over 10 days. The inhibitory effect was indicated by the appearance of fungal growth inhibition zones or by the changes in the aspect of the microfungi mycelium.

##### Determining the Antifungal Effect of Yeasts by Co-Cultivation with *Aspergillus flavus* and *Aspergillus ochraceus* Fungi 

In order to monitor the inhibition rate of mycotoxigenic fungi (*A. flavus* and *A. ochraceus*) growth, both yeast and microfungi were cultivated for 48 h/7 days on solid medium (YPGA/PDA) at room temperature. An 1 × 10^8^ cells/ mL yeast cell inoculum was prepared by resuspending the culture taken from the solid medium in sterile distilled water. A 500 µL volume of cell suspension was placed in the center of an empty Petri dish and then mixed with 19.5 mL Sabouraud medium, cooled to about 50 °C. After the homogenization and solidification of the medium, 10 µL of 1 McFarland suspension were spotted in the center of the plate. The control plates were prepared by replacing the yeast suspension with 500 µL sterile distilled water. Then, the plates were incubated at 22 °C and monitored daily until the untreated microfungi culture occupied the entire plate. The percentage of fungal growth inhibition was determined using the following formula:

(1) $$Fungal\;growth\;inhibition=\left(1-\frac{RM-RP}{RM}\right)\times \;100$$

where RM—the diameter of the fungal strain in the untreated plate; RP—the diameter of the fungal strain in the presence of yeast strains.

### 2.6. Statistical Analysis

The statistical analysis was performed using GraphPad Prism v9. For the test of growth under stress conditions and the fungal growth inhibition analysis, the results of the three replica were determined as mean ± SD and the data were analyzed using ordinary two-way ANOVA, with Tukey test as the post hoc test for multiple comparisons, with individual variances calculated for comparison between the biomass accumulation in the presence of different stress factors and the control, respectively, and between the biological activities of the yeasts and the untreated fungal strains. The significance level was set in both cases at *p* < 0.05. The level of significance was expressed as lower case letters. For all variables with the same letter, the difference between the means was not considered statistically significant. 

## 3. Results and Discussion

### 3.1. Complete Taxonomic Identification of Selected Yeast Strains

The nine yeast strains selected from the CMGB collection, and preliminary identified as belonging to *Starmerella*, *Candida*, *Metschinikowia*, and *Pichia* genera (Table 1) [40,41], were completely identified using both automatized systems and molecular methods based on a highly conserved DNA region. Both the Biolog Microbial ID System and Bruker MALDI-TOF systems are currently used for the taxonomic identification of yeasts (especially in the clinical field), with the first taking into account the metabolic profile of the strain being identified, while the second focuses on its protein profile. Using the Biolog system, an approximate taxonomic classification of the nine analyzed strains was obtained (Table 1). However, the MALDI-TOF analysis provided only one positive result (with a similarity index value > 1.5), namely, in the case of strain ST53, which was thus identified as belonging to the *Candida valida* species (Table 1). According to the studies carried out by Kačániová et al. [42] and Kurtzman et al. [43], *C. valida* is the teleomorphic form of *P. membraniefaciens*, which is why the two results do not contradict but complement each other. 

To validate the results regarding the taxonomic classification of the strains, two molecular-level analyses were used. Thus, according to Figure S1 and Table S2, it is established that the size of the amplicons obtained varies in the range of 400–625 bp and the number and size of the restriction fragments vary depending on the endonuclease used. The highest number of fragments was obtained when the amplicon was digested with *Cfo*I. A particular situation is encountered in the case of the ST19 strain, which does not present a restriction site at the level of the region of interest for *Cfo*I enzymes, or *Hae*III and *Msp*I. For the yeast species not as extensively described in the specialized literature, such as *P. (K.) ohmeri*, *C. vanderwaltii* and *M. reukaufii*, theoretical restrictions were also put in place based on the nucleotide sequences available in NCBI. Thus, a larger number of PCR-RFLP profiles could be obtained with which to make an accurate comparison. According to the results obtained up to this point, it can be considered that the PCR-RFLP analysis of the ITS1-5.8S rDNA-ITS2 region confirms the identification obtained with the help of automatic detection systems for the strains ST8.1, ST10, ST21. 1, ST21.2-*M. reukaufii*; ST19-*K. (P.) ohmeri*; ST8.2-*C. magnoliae*; and ST53-*P. membraniefaciens*. The small variations between the sizes of the fragments obtained by the present study and those described in the literature/obtained by theoretical restrictions are due to the particularities at the strain level or the amplification technique [44,45,46,47,48]. For the strains ST1 and ST12, it is found that there is a rather large difference between the sizes of the fragments obtained with the *Hae*III enzyme for the newly isolated strains and those from the specialized literature belonging to the *C. vanderwaltii* and *S. (P.) stipitis* strains. In addition, regarding the ST1 strain, differences also appear at the level of the profile generated using *Cfo*I. Taking into account the small number of profiles available in the specialized literature, we resorted to additional analyses. Thus, for the ST19, ST12, ST53, ST8.2 and ST1 strains, the ITS1-5.8S rDNA-ITS2 region was sequenced and compared to the NCBI database. Following the construction of contigs and their alignment using Blastn, their taxonomic identification was confirmed. Considering this result, the PCR RFLP profile obtained for ST1 and ST12 strains was also compared with restrictions of the sequences corresponding to the *L. thermotolerans* and *S. bombi* strains, and it was found that the differences between the sizes of the fragments decreased significantly. In the case of *L. thermotolerans* and *S. (P.) stipites*, there are no considerable differences when conventional taxonomy tests are applied [43,49], so the sequencing of the ITS1-5.8S rDNA-ITS2 region or RFLP PCR using the *Hae*III enzyme remains the only possibility to differentiate between the two species. A similar situation is also encountered in the case of the ST1 strain, initially identified as *C. vanderwaltii* and later reassigned after sequencing to the *S. bombi* species; the only difference, apart from the complete sequence of the region of interest, being the RFLP PCR profile generated using *Cfo*I and *Hae*III (Table S2).

### 3.2. Non-Pathogenic Character of the Yeast Strains

Microbial strains isolated from natural sources such as soils, plants or waters develop specific traits which help them survive in a continuously changing environment. Some of these traits might represent an advantage which might be capitalized upon in different industries or other domains associated with human activity. Sometimes, these traits, although naturally acquired when the microbial strain directly interacts with the human/animal body, might be responsible for altering its health. In this context, although the epiphytic yeasts are not generally associated with pathogenicity, except for specific species such as *Cryptococcus neoformans* and *C. gattii* [50], the first part of this study aimed to prove that the yeasts selected for this work do not pose any risk to human or animal health [51]. 

**Figure 1:**
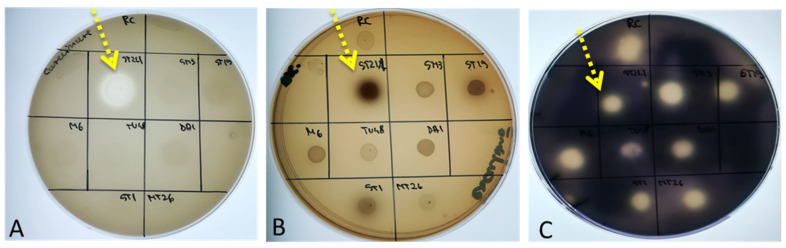
Phenotypic analysis of soluble virulence factors produced by different yeast strains ((**A**)—caseinases; (**B**)—siderophore-like compounds; (**C**)—amylolytic enzymes) (yellow arrow indicates positive result obtained for *M. reukaufii* CMGB-ST21.1 strain).

Thus, in the current investigation, a qualitative screening was undertaken to determine the production of the primary hydrolytic enzymes by yeast cells that impact the host tissues. The secretory capacity for the caseinase, amylase, gelatinase, hemolysin, phospholipase, and DN-ase strains was measured, and the results are included in Table 2. Furthermore, the sequestration ability of iron ions using siderophores as intermediate molecules was measured (Figure 1).

According to Table 2, none of the tested strains are able to produce gelatinases, phospholipases or hemolyisins, and a limited activity on the DN-ase testing culture media was recorded. Three of the four *M. reukaufii* strains secreted caseinases, and except for the *P. membranaefaciens* CMGB-ST53, all strains produced amylases. The ability of yeasts to chelate iron ions from the environment during the different phases of tissue infection is another important viulence factor. Among the main mechanisms involved is the synthesis of chelators, generally called siderophores. This phenotype was mainly observed for the *Metschinikowia* strains, and delayed action was also recorded for the *K. ohmeri* CMGB-ST19 strain.

**Figure 2:**
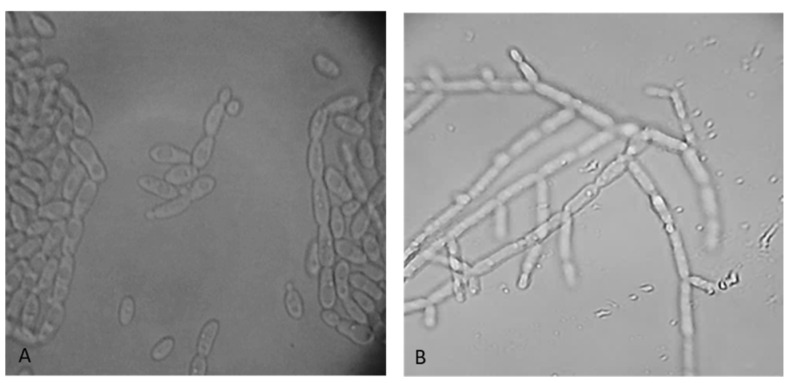
*P. membranaefaciens* CMGB-ST53 cell appearance (40×) after 24 h of incubation on YPG medium (**A**), respectively, after 3 h in the presence of fetal bovine serum (**B**).

Another hallmark of pathogenic strains is the capacity to produce biofilms. The adherence to the inert substrate is considered the first step in developing microbial biofilm. In this work, the microtitration assay with crystal violet was used to assess the total number of cells attached to the inert substratum within 72 h. The studied yeast strains did not adhere to the inert substrate, indicated by the OD values at 490 nm which were below 0.1. Another virulence characteristic of the pathogenic yeast strains is considered to be their ability to change their form from vegetative propagation by budding to a pseudohyphal/hyphal form. Although this tendency is prevalent among *Candida* strains, in the current investigation, all yeast strains were examined regardless of species. Thus, it was discovered that the majority of the strains retained their original cell shape. *P. membranaefaciens* CMGB-ST53 is the sole strain that exhibits alterations in cell morphology, with a modest percentage of pseudohyphal forms (21.81%) (Figure 2).

According to our results, none of the strains examined exhibit all virulence and pathogenicity characteristics, with the positive results suggesting the adaptability to the isolation circumstances, not their pathogenicity. Except for hemolysins, which are considered strictly involved in pathogenicity, performing screening tests such as those in this paper can be useful for selecting yeast strains with potential biocontrol applications, starting from the fact that there is a high degree of crossover between features linked with pathogenicity and antibacterial activity [32]. Thus, the yeast strains studied are safe for human health and can be employed in industries such as food and biomedicine. The strains producing amylases or caseinases are useful in the food and pharmaceutical industries as alternatives for obtaining enzymes [52,53], while strains that produce siderophore-like compounds can aid in controlling pathogenic microorganisms [54,55].

### 3.3. Yeasts Resistance to Stress Conditions

Considering the highly variable climate changes, the impact on the resistance of microbial strains to stress conditions is inevitable. This might be of benefit for the modern biotechnology since it allows for the selection of microbial strains with the naturally acquired mechanisms of high stress tolerance, which are commonly found in the industrial field. Therefore, we also tested our strains for their ability to grow in the presence of ionic, osmotic, and thermal stress.

#### 3.3.1. Influence of Thermal Stress on Biomass Accumulation

**Figure 3:**
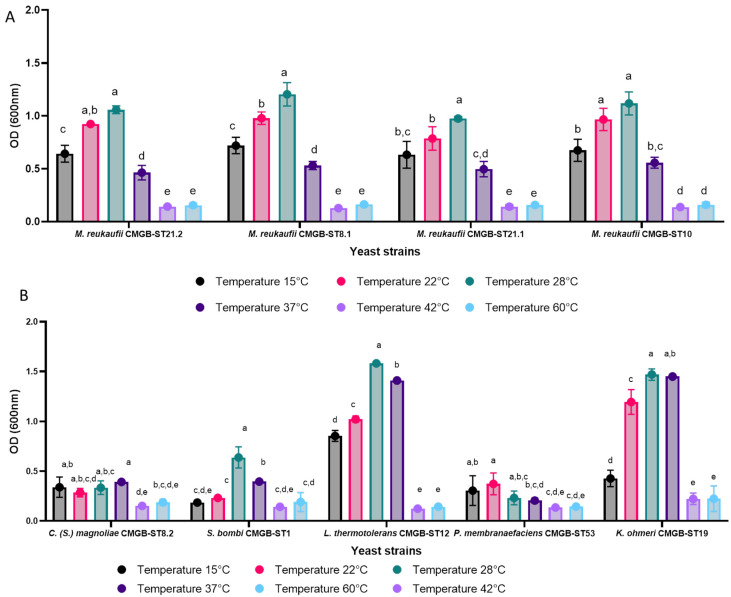
Growth of yeast strains after 24 h of cultivation at different temperature (°C) values ((**A**)—*M. reukaufii* strains; (**B**)—other yeast strains) (Tukey test, *p* < 0.05)).

The yeast strains isolated from plant surfaces thrive at temperatures between 15–28 °C, which corresponds to their source of isolation. At 42 and 60 °C, growth is hampered and OD decreases for all the strains (Figure 3). At 37 °C, for the strains *L. thermotolerans* CMGB-ST12 and *K. ohmeri* CMGB-ST19, although a reduction is observed compared to the control, the OD registered indicates their ability to tolerate higher temperatures than the ideal value indicated for yeasts in natural habitats. *L. thermotolerans*, commonly known as *Kluyveromyces thermotolerans*, is a yeast species that can tolerate temperatures as high as 35 °C [56]. Resistance to temperatures over this value has only been observed in investigations that included other causes of stress, such as the use of natural bacterial antagonists to simulate competition between microorganisms inhabiting distinct ecological niches. Zhou et al. [57] found that heat stress resistance increased when strains were cultivated at higher temperatures. Although the mechanism of resistance to thermal, ethanol, or oxidative stress in this species is still not fully understood, the biotechnological applications are important; the members of this species are being studied for potential use in producing acidic drinks and wines, and, also, *L. thermotolerans* can improve the flavor and quality of the drinks produced [56,57,58]. The strain *K. ohmeri* CMGB-ST19 showed a small increase at higher temperatures, indicating the need for a longer incubation period to study its response to heat stress. *M. reukaufii*, along with *Metschnikowia gruessii*, are the best known and most common yeasts isolated from the microbial communities associated with nectar-producing flowers [59], which show resistance to numerous stress factors, with the ecological niche being quite strict and exerting primarily osmotic stress due to the high concentration of sugars in the composition of the nectar. Until now, most studies on the species *M. reukaufii* are superficial and require more attention, focusing mostly on the investigation of the dynamics of the microbial population in such communities and the impact of anthropization on this ecological niche [60,61]. In this investigation, four strains of the species *M. reukaufii* were recovered from the surface of a large-leaved lupine flower, black elderberry leaves, and a rapeseed flower. Figure 3 indicates that *M. reukaufii* strains thrive at 28 °C, but can also develop effectively at lower or higher temperature levels. None of the four strains withstand temperatures above 37 °C, and the growth observed was limited.

#### 3.3.2. Influence of Ionic and Osmotic Stress on Biomass Accumulation

**Figure 4:**
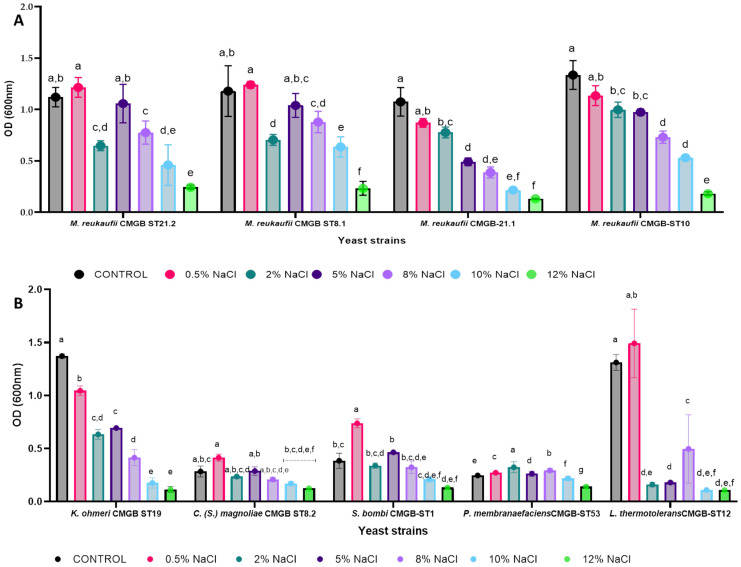
Growth of yeast strains after 24 h of cultivation at different NaCl (%) concentrations ((**A**)—*M. reukaufii* strains; (**B**)—other yeast strains) (Tukey test, *p* < 0.05).

The presence of NaCl in the culture media causes yeast cells to experience both osmotic and ionic stress; therefore, they must tolerate this compound while maintaining their biotechnological potential. In the current investigation, the yeasts proliferation ability after exposure to different NaCl concentrations was assessed by determining the OD fluctuation 24 h after inoculation (Figure 4). Although the presence of NaCl in the culture media in general reduces the ability of yeasts to grow, in the case of *S. bombi* CMGB-ST1 it seems that the growth is stimulated at 0.5%. Also, the maximum NaCl concentration at which growth is observed among the nine strains studied is 8%, with the strains *M. reukaufii* CMGB-ST21.2, *M. reukaufii* CMGB-ST.8.1, and *M. reukaufii* CMGB ST10 able to survive even 10% concentrations. When the growth rates of the four *M. reukaufii* strains are compared, it turns out that the OD of the *M. reukaufii* strains decreases as the NaCl concentration increases.

**Figure 5:**
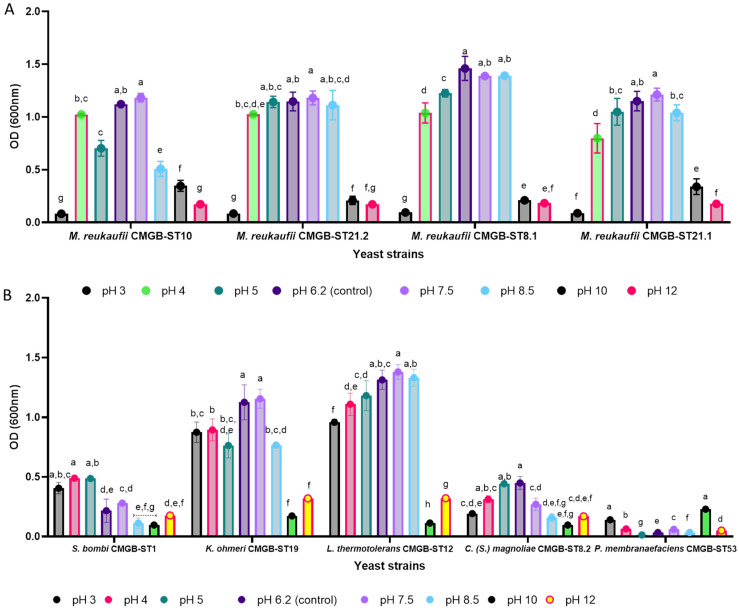
Growth of yeast strains after 24 h of cultivation in presence of different pH values ((**A**)—*M. reukaufii* strains; (**B**)—other yeast strains) (Tukey test, *p* < 0.05).

In industrial processes, the pH variation represents a stress condition for the microorganisms. Yeasts thrive at slightly acidic pH values (~6.2), but have developed defense mechanisms to withstand lower values. These mechanisms include changing cell membrane permeability, overexpressing genes involved in proton pump synthesis, and consuming protons entering the intracellular environment through metabolic processes. The accumulation of organic acids in the culture medium causes a fall in the pH value, which is frequently observed in the food industry [60]. A restricted number of yeast strains are able to develop at alkaline levels of pH, with strains from the genus *Candida* notably distinguished [61]. In the present study, the growth rate of yeast strains was determined at pH values in the range of 3–12 at 24 h by monitoring the variation in OD_600nm_. Thus, the strains *L. thermotolerans* CMGB-ST12 and *K. ohmeri* CMGB-ST19 tolerate all the pH values to which they were exposed (both strongly acidic and strongly alkaline). For the *C. magnoliae* strain, CMGB-ST8.2 the optimal pH range is 4–7.5, with a growth maximum recorded at pH 6.2. In the case of the strain *S. bombi* CMGB-ST1, the preference for the environment with more acidic pH values is found, with the maximum OD values being recorded at pH 4 and pH 5 (Figure 5).

The strains of *M. reukaufii* analyzed in this study have an optimal pH range between 4 and 8.5, being comparable to OD. It is found that the strain *M. reukaufii* CMGB ST8.1 records the highest OD value at pH 6.2, while the other strains develop optimally at pH 7.5. Also, in Figure 5, it can be observed that the strain *M. reukaufii* CMGB-ST10 has a different behavior, registering a decrease in OD at pH 6.2 compared to the neighboring values. In this case, it can be assumed that this is a result of a specific mechanism of adaptation to pH variations that most likely focuses on the activation of the transcription of some genes involved in the specific cellular response to acidic pH values. Therefore, most of the yeast strains tolerated the pH variation in the range of 4–7.5, and the tolerance to the ionic stress induced by strongly acidic or strongly alkaline pH depends both on the species from which the yeast strain originates and the species it belongs to. The exposure of the nine yeast strains to stress conditions indicates that most of them can be taken into consideration for further studies aimed at establishing their potential use for industrial applications, although their tolerance differs depending on the nature of the stress factor.

### 3.4. Antimicrobial Activity of Epiphytic Yeasts

Many of the epiphytic microorganisms and yeasts, in particular, exhibit antimicrobial properties and can be successfully used as biocontrol agents. In this study, we investigated both their potential use as antimicrobial agents for biomedicine and for the food industry, as well for their usage as biocontrol agents against phytopatogenic fungi.

#### 3.4.1. Anti-Candida Potential

For their antimicrobial activity against *Candida* strains, two protocols were tested. The first involved determining the inhibitory effect based on competition for nutritive substrates, while the second was based on the antimicrobial activity at low pH values.

According to Table 3, it is found that the antimicrobial action of the tested strains based on iron ion competition is present only in a limited number of strains. Among these, the *M. reukaufii* CMGB-ST8.1 strains stand out; *C. magnoliae* CMGB-ST8.2; *K. ohmeri* CMGB-ST19; *M. reukaufii* CMGB-ST21.2; *M. pulcherrima* CMGB-M3 and *P. membranaefaciens* CMGB-ST53, whose antimicrobial activity was evident in the presence of *C. parapsilosis*, as well as CBS 604 and *C. krusei* CMGB94. A positive result was also recorded when the pathogenic strain *C. catenulata* CMGB-Y7 was used as a potentially sensitive substrate. In this case, the antimicrobial action was highlighted for the strains *L. thermotolerans* CMGB-ST12 and *K. ohmeri* CMGB-ST19 after 7 days of incubation.

Except for the strains *P. membranaefaciens* CMGB-ST53 and *L. thermotolerans* CMGB-ST12, all other strains, according to the results described above regarding the pathogenicity profile, are able to produce siderophore-like compounds that can represent an advantage for the producing yeast strain in the competition for the substrate that best facilitates the accumulation of iron from the environment. In the case of *Metschnikowia* species (*M. sinensis*, *M. shaxiensis* and *M. fructicola*) [62], the antimicrobial action is based on the competition for iron ions in the environment by the excreted pulcherrimic acid, which, in the presence of iron, forms the reddish-brown pigment named pulcherrimine. The retained iron ions from the culture media become inaccessible to competing strains. Although, until recently, *M. reukaufii* was not investigated from this point of view, due to its ability to synthesize siderophore-like compounds, there is a possibility that the action of the two strains included in this species is due to the synthesis of such compounds, which requires further investigation. The strain *P. membranaefaciens* CMGB-ST53, although it does not secrete siderophor-like compounds, shows antimicrobial activity against *C. parapsilosis* CBS604 and *C. krusei* CMGB94 strains. Members of these species, although they develop slightly reddish-brown colonies after longer cultivation on agar medium, are not included among the strains whose main mechanism of antimicrobial action is competition for the nutrients. *Pichia* species are mostly known for their ability to produce killer toxins that act similarly to the killer toxin of *S. cerevisiae*. The presence of antimicrobial activity at pH 6.0 is atypical for members of this species; with the optimal action being recorded at acidic pH values [63]. In the case of *L. thermotolerans*, the studies on antimicrobial activity are not in-depth, being based on its ability to inhibit the production of mycotoxigenic compounds by *Aspergillus* and *Fusarium* due to the synthesis of volatile organic compounds that inhibit both mycelial growth and mycotoxin production [64].

The antimicrobial activity at low pH values was determined using the K (killer) medium originally described for *S. cerevisiae.* The yeast strain with antimicrobial potential was inoculated as a spot, starting from a suspension of known cellular density on the medium K containing methylene blue, which allows for the appearance of an inhibition zone and potential cell changes.

According to Table 4, although the strain *P. membranaefaciens* CMGB-ST53 inhibited the growth of strains *C. parapsilosis* CBS604 and *C. krusei* CMGB94 on YMA medium, this capacity was not maintained at acidic pH values. The strain *S. bombi* CMGB-ST1 is also noteworthy, inhibiting both the growth of the collection strains *C. krusei* CMGB94 and *C. albicans* ATCC10231, and the pathogenic strains *C. parapsilosis* CMGB-Y3 and *C. albicans* CMGB-Y12. Regarding the inhibited pathogenic strains, it is noted that the strain *S. bombi* CMGB-ST1 has satisfactory action from the first three days of incubation, which denotes the importance of studying this strain, considering the fact that it is one of the few strains that inhibited the growth these pathogens. Except for the *K. ohmeri* CMGB-ST19 strain, for the other yeast strains, it is shown that they mainly inhibit the two strains of *C. krusei* and present a limited effect on other *Candida* strains. *K. ohmeri* CMGB-ST19 is part of a species described as having antimicrobial potential based on the synthesis of killer toxins encoded by nuclear genes. However, the mechanism of action is not fully described and the number of pathogenic strains on which such studies have been carried out is limited. In the present study, it was outlined that this strain has an inhibitory effect on the strains of the species *C. parapsilosis*, *C. krusei* and *C. albicans*, acting both on pathogenic strains and on laboratory strains.

#### 3.4.2. Anti-Bacterial Potential

In general, the antimicrobial activity of the yeasts against other yeasts or filamentous fungi is focused on and emphasized, and only few recent investigations have revealed their ability to produce secondary metabolites that limit bacterial growth [65], with obvious importance for the biomedical field for limiting the bacterial infections, and for industry in wine fermentation, where some groups of bacteria can affect the quality of the final product [66]. The fundamental issue is the inability to provide adequate culture conditions for both bacterial and yeast strains. The current study aims to choose an optimal culture medium for the growth of the two types of microorganisms, while also qualitatively evaluating the antibacterial capability of the nine epiphytic yeast strains. The protocol used to determine the antibacterial activity is similar to the one described for testing against *Candida* strains, with the adjustment that in this case, the bacterial strains were inoculated on several medium variants, namely: NA, BHI, MH, YGSA. Except for the YGSA medium, the other culture media did not allow for the simultaneous growth of both bacteria and yeasts. Although several studies on the antibacterial activity of yeasts recommend the use of media such as NutrientAgar or Mueller–Hinton, they do not aim to perform large-scale screening tests, but focus on testing the antimicrobial compound produced by yeasts [65,66,67]. Thus, these media formulas lend themselves to the following stages of such a study, which involve the purification of the compound with antimicrobial activity and the determination of the value of the minimum inhibitory concentration in accordance with the Clinical and Laboratory Standards Institute (CLSI) guidelines pertinent to the isolation year [68]. The YGSA medium is described by Acuña-Fontecilla et al. [38] as the optimal medium variant for screening tests on the antibacterial activity of yeast strains from natural isolates. This culture medium contains the nitrogen source, the carbon source, and NaCl which, on one hand, simulates the osmotic stress conditions common in the various ecological niches and, on the other hand, stimulates the antimicrobial activity of several yeast species. Also, to ensure a better observation of the inhibition halos, the culture medium contains methylene blue. However, unlike the other variations studied, the medium has an acidic pH, which promotes yeast strain growth and, implicitly, antibacterial action. Although bacterial strains such as *S. aureus*, *B. cereus*, and *L. monocytogenes* have an ideal pH range for growth of 4–5 to 9.8, such a pH value is difficult to endure, especially when combined with NaCl, which adds to the stress. The antimicrobial activity test did not allow for the determination of inhibition halos for these strains, since they demonstrated delayed and uneven growth.

According to Table 5, the antibacterial activity of the tested yeast strains is mainly exercised against Gram-negative bacteria, such as *E. coli* ATCC25922 and *P. aeruginosa* ATCC27853. The best results were obtained in the case of *S. bombi* CMGB-ST1 and *C. magnolia* CMGB-ST8.2. For testing the antibacterial activity against Gram-positive bacterial strains, using a culture medium such as Mueller–Hinton or Nutrient Agar is recommended. However, because it is not an optimal growth medium for yeasts, it is necessary to know the antimicrobial mechanisms involved and, for those based on secondary metabolites with antimicrobial properties, to purify them and directly test their inhibitory activity. Although the biotechnological potential of the *S. bombi* species is not well described, other *Starmerella* clade members are known for their ability to produce secondary metabolites with antimicrobial properties. As an example, the sophorolipids produced by *S. bombicola* ATCC22214 are efficient against the *Bacillus subtilis, Staphylococcus aureus, E. coli* and *P. aeruginosa* bacterial strains [69].

#### 3.4.3. Practical Applications for Developing Probiotic Products

For establishing the practical use of the tested strains as functional microorganisms for the improvement of humans’ health, we tested their ability to release the butyric acid which acts as a mediator of the expression of many genes involved in basic metabolism, as an antioxidant, and as an immune response modulator. Furthermore, this chemical stimulates colonocyte activity and benefits gut microbiota [70]. Simultaneously, it is also important that any microorganisms used for their antimicrobial activity, regardless of the research field being discussed, have a limited or non-existing impact on the natural gut microbiota.

First, the lipolytic activity of the yeast strains was evaluated by cultivation on YPTA medium containing tributyrin; the ability of the strains to hydrolyze tributyrin being is demonstrated by the development of halos surrounding the culture spots, which occur from the cleavage of the ester bonds in the tributyrin structure and the release of butyric acid. According to Table 6, almost all the strains exhibited lipolytic activity, but the best results were described for the *S. bombi* CMGB-ST1 strain, for which a positive reaction was described at all tested temperature values. Although less active, *L. thermotolerans* CMGB-ST12 hydrolyzed tributyrin at 28 °C and 37 °C (Table 6).

According to Table 7, the *M. reukaufii* strains and the strain *C. magnoliae* CMGB-ST8.2 inhibited the growth of the *S. boulardii* CMGB-S and *K. marxianus* CMGB159, but not the growth of *K. lactis* CMGB112 or lactic acid bacteria. In the case of these strains, the antimicrobial activity is most likely due to a synergistic effect between the antimicrobial activity of the tested strains and the exposure of the probiotic strains to acidic pH values. Although it is currently used as an oral probiotic, *S. boulardii* grows optimally when the pH is 6.8 and long-term exposure to acidic pH (below 5) causes a decrease in cell viability, which explains its use together with pharmaceutical excipients that protect it against the acidic pH in the stomach [71]. Lately, other yeasts belonging to *Kluyveromyces*, *Yarrowia* and *Torulaspora* were described as microorganisms with potential health benefits, some being included in the “Qualified Presumption of Safety” list approved by the European Food Safety Authority. A study conducted by Agarbati et al. [25] showed that *L. thermotolerans* strains isolated from grapes or wine from Italy might represent a valid alternative for probiotics development, and recommend further investigation regarding their health-promoting efficacy. Similar results were also described by Fernandez-Pacheco et al. [72], who demonstrated that *L. thermotolerans* from a winery presents prebiotic metabolism and a higher variability of enzymatic activities associated with health-promoting ability. For the *P. membranaefaciens* species, it was demonstrated that it presents a number of desirable metabolic features (such as the ability to synthesis vitamins of the B-complex) for including it in the range of potential probiotic yeast species [73].

### 3.5. Epiphytic Yeasts as Biocontrol Agents

In addition to their applications in the biomedical field, yeasts are also characterized as agents involved in the biocontrol of phytopathogenic filamentous fungi, which are known as the main contaminants of grains when stored in silos. The last part of the present study aimed to demonstrate the antifungal potential of the epiphytic yeasts against common fungal strains known to be involved in crop contamination. The assessment involved both observing the direct interaction between yeasts and fungi and their potential for inhibiting the mycelial growth of mycotoxigenic fungi.

The initial stage in determining the antifungal ability of yeast strains was to conduct a qualitative assessment of their direct contact with seven filamentous fungal strains belonging to *B. cinereea*, *R. solani*, *A. ochraceus*, and *A. flavus* species, using agarized Sabouraud media. The inhibitory impact of the tested yeasts is determined by the emergence of zones where the mycelial growth is slowed down in the vicinity of the yeast streak.

**Figure 6:**
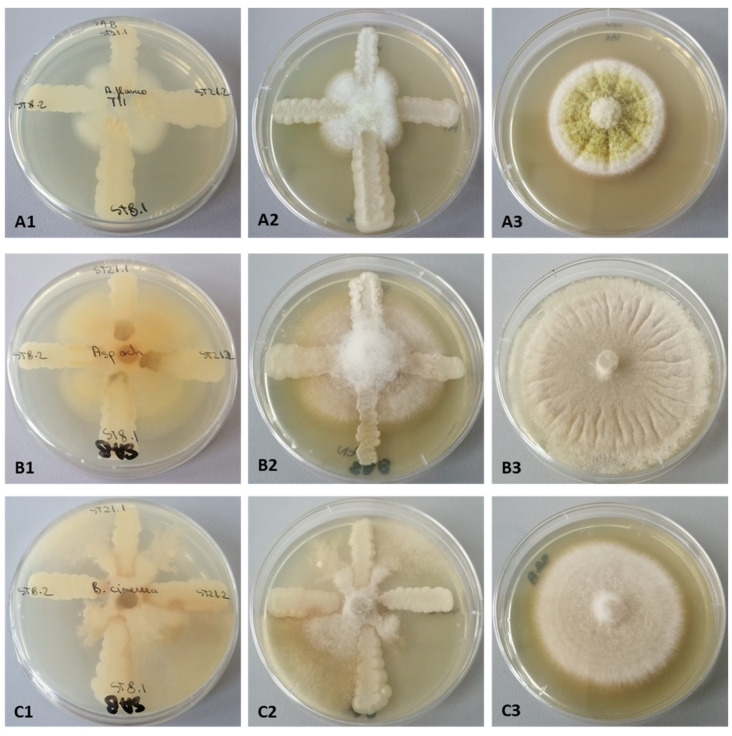
The interaction between the yeast strains and *A. flavus* T11—(**A1**–**A3**); *A. ochraceus*—(**B1**–**B3**); *B. cinereea*—(**C1**–**C3**) (1;2—yeasts-fungi interaction; 3—control).

The antifungal potential of yeast strains varies both depending on the species to which it belongs and on the fungus used for the determination (Figure 6). Thus, in Table 7, it is observed that the strain *C. magnoliae* CMGB-ST8.2 is the only strain in which the zone of inhibition was observed for all seven strains of filamentous fungi tested.

**Figure 7:**
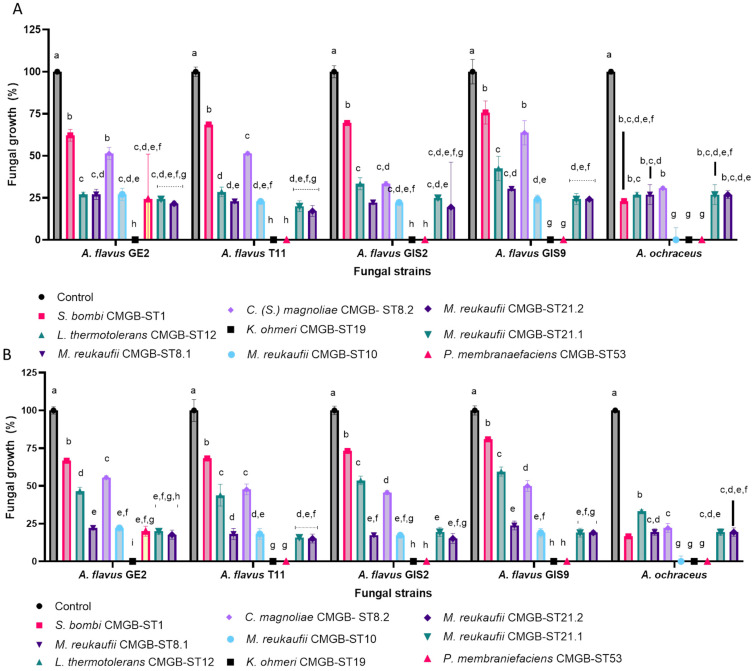
Determination of the inhibition percentage of mycelial growth in the case of strains of filamentous fungi belonging to the genus *Aspergillus* after 5 days of incubation (**A**) and 8 days of incubation (**B**) (Tukey test, *p* < 0.05).

The quantitative evaluation of the ability of yeast strains to inhibit the mycelial growth of filamentous fungal strains involved their co-cultivation on Sabouraud medium for 8 days and the periodic measurement of the diameter of the fungal colony. The results were quantified at 5 and 8 days (Figure 7, Table 8) by determining the percentage of inhibition in reference to the untreated control with the yeast suspension. According to Figure 7, the best inhibitory effect was observed for *K. ohmeri* CMGBST19, which completely inhibited growth of the five fungi strains starting with the fifth day of incubation. Good results were also obtained for *P. membranaefaciens* CMGB-ST53, for which the inhibition percentage is included between 80% and 100%. *L. thermotolerans* CMGB-ST12 also presented important antifungal activity, with the inhibition percentage being maintained around 50% regardless of the fungal strain tested or the incubation time. Recent studies recommend *L. thermotoloerans* as an important yeast species for improving wine quality due to its ability to secrete lactic acid, terpenes, and isobutyrat, or to reduce the acetic acid or acetaldehyde concentrations [74]. Apart from these traits, *L. thermotolerans* strains also exhibit antifungal activity against *Aspergillus parasiticus, Penicillium verrucosum* and *F. graminearum*, inhibiting through the volatile organic acids secreted both their growth and the ability to secrete mycotoxins [64]. Also, it exhibits high antifungal activity against the *Aspergillus carbonarius* and *A. niger* ochratoxin A-producing strains from grape berries [75,76].

## 4. Conclusions

Food and crop contamination with microorganisms resistant to natural or man-made antimicrobial agents, represent a growing problem at an international level, with high impact on human health. The epiphytic yeasts adapt to environmental conditions and compete with other microorganisms from the same habitat, thus representing an interesting resource which might be capitalized upon for agricultural, industrial and biomedical applications. The nine studied yeast strains, belonging to the *Starmerella*, *Candida*, *Metschinikowia*, *Lachancea*, *Kodamaea* and *Pichia* genera, did not exhibit pathogenicity and virulence traits, registered high cell growth under stress conditions, showed good anti-*Candida* and antibacterial activity and a high inhibition percentage of the mycelial development of phytopathogenic fungi, hydrolyzed the tributyrin, and are susceptible to co-cultivation with probiotic yeast strains. Overall, the results indicate the strains *K. ohmeri* CMGB-ST19 and *M. reukaufii* CMGB-ST21.2 as the most promising. We also mention that this is one of the few studies revealing the antimicrobial activity of a *L. thermotolerans* strain. We can estimate that, in conclusion, these characteristics recommend them for development of new strategies aimed at preventing and combating the spreading of food microbial contaminants. However, further investigations will aim to test a wider range of human pathogenic bacteria and yeasts, with a focus on the mechanism of antimicrobial action and in vivo studies, and molecular studies for the determination of the yeast effect in the inhibition of mycotoxin production by phytopathogenic fungi.

## Figures and Tables

**Table 1:** Yeast strains and taxonomic identification using Biolog Microbial ID System; Bruker MALDI-TOF and molecular methods.

Strain	Biolog Microbial ID System	Bruker MALDI-TOF	PCR-RFLP Profile on of ITS1-5.8SrDNA-ITS2 Region	Sanger Sequencing of ITS1-5.8SrDNA-ITS2 Region
ST1	*C. vanderwaltii*	No id	*C. vanderwaltii/S. bombi*	*S. bombi*
ST8.1	*M.* *reukaufii*	No id	*M. reukaufii*	Nd
ST8.2	*Candida magnoliae*	No id	*C. magnoliae*	*S. (C.) magnoliae*
ST10	*M. reukaufii*	No id	*M. reukaufii*	Nd
ST12	*Scheffersomyces (Pichia) stipitis*	No id	*S.(P.) stipites/L. thermotolerans*	*L. thermotolerans*
ST19	*K. (P.) ohmeri*	No id	*K. (P.) ohmeri*	*K. (P.) ohmeri*
ST21.1	*M. reukaufii*	No id	*M. reukaufii*	Nd
ST21.2	*M. reukaufii*	No id	*M. reukaufii*	Nd
ST53	*P. membraniefaciens*	*C. valida*	*P. membraniefaciens*	*P. membraniefaciens*

Nd—not determined.

**Table 2:** Evaluation of the ability to produce soluble virulence factors.

Strain	Caseinases	Amylases	Gelatinases	Hemolysins	Phospholipases	Siderophores	DN-Ase
*S. bombi* CMGB-ST1	−	W	−	−	−	D	−
*L. thermotolerans* CMGB-ST12	−	+	−	−	−	−	D
*M. reukaufii* CMGB-ST8.1	+	+	−	−	−	+	−
*C. magnoliae* CMGB-ST8.2	−	+	−	−	−	W	−
*M. reukaufii* CMGB-ST10	+	+	−	−	−	+	W
*K. ohmeri* CMGB-ST19	−	+	−	−	−	D	D
*P. membranaefaciens* CMGB-ST53	−	−	−	−	−	−	−
*M. reukaufii* CMGB-ST21.1	+	+	−	−	−	+	−
*M. reukaufii* CMGB-ST21.2	−	+	−	−	−	+	D

Legend: “−” negative results; “+” positive results, “D”—delayed positive results (the positive result appeared after the recommended time of determination); “W”—weak positive results (the positive result is recorded on the day intended for monitoring, but its intensity is considered lower than for the complete positive result).

**Table 3:** Antimicrobial activity based on iron ions competition against *Candida* strains.

Strain		*C. parapsilosis* CBS 604	*C. parapsilosis* CMGBY3	*C. krusei* CMGB 94	*C. krusei* CMGB-Y8	*C. catenulata* CMGB-Y7	*C. albicans* ATCC10231	*C. albicans* CMGB-Y12
*S. bombi* CMGB-ST1	3 days	−	−	+	−	−	−	−
	7 days	−	−	−	−	−	−	−
*L. thermotolerans* CMGB-ST12	3 days	−	−	−	−	−	−	−
	7 days	−	−	−	−	+	−	−
*M. reukaufii* CMGB-ST8.1	3 days	−	−	−	−	−	−	−
	7 days	+	−	+	−	−	−	−
*C. magnoliae* CMGB- ST8.2	3 days	−	−	+	−	−	−	−
	7 days	+	−	+	−	−	−	−
*K. ohmeri* CMGB-ST19	3 days	−	−	−	−	−	−	−
	7 days	++	−	−	−	+	−	−
*P. membranaefaciens* CMGB-ST53	3 days	−	−	−	−	−	−	−
	7 days	++	−	+	−	−	−	−
*M. reukaufii* CMGB-ST21.2	3 days	−	−	−	−	−	−	−
	7 days	++	−	+	−	−	−	−
*M. reukaufii* CMGB-ST21.1	3 days	−	−	−	−	−	−	−
	7 days	+	−	−	−	−	−	−

Caption: “−” lack of halo or the halo size < 2 mm; “+” 2–3 mm halo with medium luminous intensity; “++” halo of 2–3 mm with high luminous intensity.

**Table 4:** The antimicrobial activity in the presence of low pH values against *Candida* strains.

Strains		*C. parapsilosis* CBS 604	*C. parapsilosis* CMGBY3	*C. krusei* CMGB94	*C. krusei* CMGB Y8	*C. catenulata* CMGB-Y7	*C. albicans* ATCC 10231	*C. albicans* CMGB-Y12
*S. bombi* CMGB-ST1	3 days	−	++	+	−	−	+	+
	7 days	−	++	++	−	−	+	++
*L. thermotolerans* CMGB-ST12	3 days	−	−	−	−	−	+	+
	7 days	−	−	−	−	−	+	−
*M. reukaufii* CMGBST8.1	3 days	−	−	+	++	−	+	−
	7 days	−	−	+	++	−	+	−
*C. magnoliae* CMGB- ST8.2	3 days	−	−	+	++	−	−	−
	7 days	−	+	+	+	−	−	−
*M. reukaufii* CMGB-ST10	3 days	−	−	+	++	−	+	−
	7 days	−	−	+	++	−	+	−
*K. ohmeri* CMGB-ST19	3 days	−	−	+	+	−	+	+
	7 days	−	+	+	+++	−	+	−
*P. membranaefaciens* CMGB-ST53	3 days	−	−	−	−	−	−	−
	7 days	−	−	−	−	−	−	−
*M. reukaufii* CMGB-ST21.1	3 days	−	−	+	+	−	−	−
	7 days	−	−	+	+	−	−	−
*M. reukaufii* CMGB-ST21.2	3 days	−	−	+	++	−	+	−
	7 days	−	−	+	++	−	+	−

Caption: “−” lack of halo or the halo size < 2 mm; “+” 2–3 mm halo with medium luminous intensity; “++” halo of 2–3 mm with high luminous intensity; “+++” 5–6 mm halo.

**Table 5:** Qualitative evaluation of the antibacterial activity of yeast strains (YGSA medium).

Strains	*S. typhimurium* ATCC14028	*L. monoxytogenes* CMGB333	*B. cereus* CMGB 53-100	*E. coli* ATCC 259-22	*P. aeruginosa* ATCC 27853	*S. aureus* ATCC6538
*S. bombi* CMGB-ST1	−	S	S	+	+	S
*L. thermotolerans* CMGB-ST12	−	S	S	−	−	S
*M. reukaufii* CMGB-ST8.1	−	S	S	W	W	S
*C. magnoliae* CMGB-ST8.2	W	S	S	+	+	S
*M. reukaufii* CMGB-ST10	−	S	S	−	−	S
*K. ohmeri* CMGB-ST19	−	S	S	−	−	S
*P. membranaefaciens* CMGB-ST53	−	S	S	−	−	S
*M. reukaufii* CMGB-ST21.1	−	S	S	−	++	S
*M. reukaufii* CMGB-ST21.2	−	S	S	−	+	S

Caption: “W”< 2 mm halo “+” 2–3 mm halo; “++” halo > 4 mm; “−” lack of halo; S—the bacterial strain did not grow evenly on the surface of the culture media;.

**Table 6:** Qualitative evaluation of the yeasts’ ability to hydrolyze tributyrin.

Strains	20 °C		28 °C		37 °C	
	4 Days	7 Days	4 Days	7 Days	4 Days	7 Days
*S. bombi* CMGB-ST1	+	++	+++	+++	+	+
*L. thermotolerans* CMGB-ST12	W	+	+	+	+	+
*M. reukaufii* CMGB-ST8.1	−	W	+	+	−	−
*C. magnoliae* CMGB-ST8.2	−	W	+	+	−	−
*M. reukaufii* CMGB-ST10	W	+	+	+	W	+
*K. ohmeri* CMGB-ST19	W	+	+	+	+	+
*P. membranaefaciens* CMGB-ST53	W	+	+	+	−	−
*M. reukaufii* CMGB-ST21.1	W	+	+	+	W	−
*M. reukaufii* CMGB-ST21.2	W	+	+	+	W	−

Caption: “−” negative result; “W” positive result-1 < halo < 1.2, “+” positive result 1.2 < halo < 1.5 “++” positive result 1.5 < halo < 2, “+++” positive result 2 < halo < 3.5.

**Table 7:** The antimicrobial activity against probiotic microorganisms.

Strains	*S. boulardii* CMGB-S		*K. marxianus* CMGB159		*K. lactis* CMGB112		*S. salivarius* ATCC 19258	*Lb. acidophilus* ATCC 4356
	3 Days	7 Days	3 Days	7 Days	3 Days	7 Days	1 Day	1 Day
*S. bombi* CMGB-ST1	−	−	−	−	−	−	−	−
*L. thermotolerans* CMGB-ST12	−	−	−	−	−	−	−	−
*M. reukaufii* CMGBST8.1	+	+	+	++	−	−	−	−
*C. magnoliae* CMGB-ST8.2	++	++	++	++	−	+	−	−
*M. reukaufii* CMGB-ST10	+	++	+	++	−	−	−	−
*K. ohmeri* CMGB-ST19	−	−	−	−	−	−	−	−
*P. membranaefaciens* CMGB-ST53	−	−	−	−	−	−	−	−
*M. reukaufii* CMGB-ST21.1	+	−	+	−	−	−	−	−
*M. reukaufii* CMGB-ST21.2	++	++	+	−	−	−	−	−

Caption: “−” lack of halo; “+” 2–3 mm halo with medium luminous intensity; “++” 2–3 mm halo with high luminous intensity.

**Table 8:** Qualitative investigation of the antifungal property of yeast strains (7 days; room temperature; Sabouraud medium) (“+” fungal growth/sporulation inhibition; “−” no fungal growth/sporulation inhibition.

Strains	*B. cinereea*	*A. ochraceus*	*R. solani*	*A. flavus* T11	*A. flavus* GE2	*A. flavus* GIS2	*A. flavus* GIS9
*S. bombi* CMGB-ST1	+	+	−	+	+	−	+
*L. thermotolerans* CMGB-ST12	+	+	−	−	−	−	+
*M. reukaufii* CMGB-ST8.1	+	−	−	+	+	+	+
*C. magnoliae* CMGB-ST8.2	+	+	+	+	+	+	+
*M. reukaufii* CMGB-ST10	+	+	−	−	−	−	+
*K. ohmeri* CMGB-ST19	+	+	−	+	+	−	+
*P. membranaefaciens* CMGB-ST53		+	−	+	+	+	+
*M. reukaufii* CMGB-ST21.1	+	+	−	+	+	−	+
*M. reukaufii* CMGB-ST21.2	+	+	−	+	+	+	+

## Data Availability

All data are available on request to the corresponding author.

## Supplementary Materials

The following supporting information can be downloaded at: https://www.mdpi.com/article/10.3390/life14091087/s1, Table S1: Yeast strains and isolation sources; Figure S1: PCR-RFLP profile of the ITS1-5.8S-ITS2 region obtained for the strains isolated from the plant surface; Table S2: Size of amplicons and restriction fragments obtained for the ITS1-5.8S-ITS2 region for the epiphytic yeast strains and strains belonging to species of interest described in the literature.
