# Supplementary material for: Epiphytic Yeasts from South Romania for Preventing Food Microbial Contamination
Source: Life (Basel). 2024 Aug 29;14(9):1087. doi: 10.3390/life14091087 (PMC11433553; doi:10.3390/life14091087)
Supplement: Supplementary file 1 [file life-14-01087-s001.zip › life-3131830-supplementary.pdf]

Article

Epiphytic yeasts from South Romania for preventing food microbial contamination

Viorica Maria Corbu [1,2], Andreea Ștefania Dumbravă [3,4], Irina Gheorghe-Barbu[2,4] and Ortansa Csutak [1,2,*]

[1] Department of Genetics, Faculty of Biology, University of Bucharest, Aleea Portocalelor 1-3, 060101, Bucharest, Romania; V-M.C. viorica-maria.corbu@bio.unibuc.ro; O. C. ortansa.csutak@bio.unibuc.ro;
[2] Research Institute of University of Bucharest (ICUB), B.P. Hasdeu Street 7, 050568, Bucharest, Romania;
[3] Department of Technological Irradiation (IRASM), Horia Hulubei National Institute of Physics and Nuclear Engineering–IFIN-HH, Măgurele, Romania; A-S.D. andreeadum29@gmail.com ;
[4] Department of Microbiology and Botany, Faculty of Biology, University of Bucharest, Aleea Portocalelor, no1-3, 060101, Bucharest, Romania; I.G.B. irina.gheorghe@bio.unibuc.ro
*Correspondence: ortansa.csutak@bio.unibuc.ro;

Supplementary materials

Tabel S1. Yeast strains and isolation sources

Isolation source	Yeast strains
Peony flower (*Paeonia peregrina*)	CMGB-ST1
Jasmine flower (*Jasminum officinale*)	CMGB-ST12
Large-leaved lupine flower (*Lupinus poliphyllus*)	CMGB-ST8.1
	CMGB-ST8.2
Black elderberry leaves (*Sambucus nigra*)	CMGB-ST10
Sweetness rose flower (*Rosa damascena*)	CMGB-ST53
	CMGB-ST19
Oilseed rape flower (*Brassica napus*)	CMGB-ST21.1
	CMGB-ST21.2

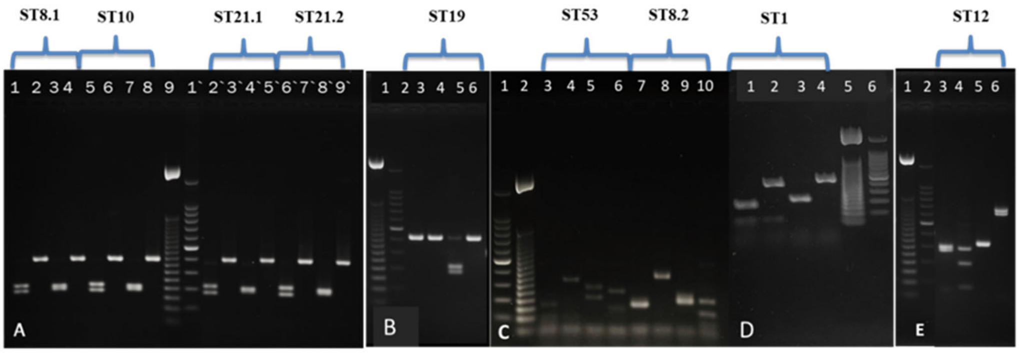

Figure S1: PCR-RFLP profile of the ITS1-5.8S-ITS2 region obtained for the strains isolated from the plant surface A:1-4-ST8.1; 5-8-ST10; 2`-5-ST21.1; 6`-9`-ST21.2;B:3-6-ST19; C: 3-6-ST53; 7-10-ST8.2; D:1-4-ST1; E:3-6-ST12). For each strain the restriction fragments were loaded according to the order: *Cfo* I; *Hae* III; *Hinf* I; *Msp* I. A-9; B-1; C-2; D-5; E-1- BenchTop-50-bp-DNA Ladder (Promega) A-1`; B-2; C-1; D-6; E-2- 100-bp DNA Ladder (ThermoFisher Scientific);

Table S2. Size of amplicons and restriction fragments obtained for the ITS1-5.8S-ITS2 region for the epiphytic yeast strains and strains belonging to species of interest described in the literature

Strain	Amplicon (bp)	*Cfo*I (bp)	*Hae*III (bp)	*Hinf*I (bp)	*Msp*I (bp)	Reference
ST8.2	400	200;200	350	200; 200	125;200	
C. magnoliae	400	43;56;114; 132	28;100;217	8;153;184	ND	Arroyo-López et al., 2006
C. magnoliae	425	190;200	140;285	200;225	ND	Arroyo-López et al., 2006
C. magnoliae	425	190;200	140;285	200;225	ND	Arroyo-López et al., 2006
ST12	625	275;300	75;200;300	300;300	600	
S. stipitis CBS 6054	628	48; 273;297	135;493	307;313	ND	Pham et al., 2011
S. stipitis CBS 5773	650	285;300	140;490	310;310	ND	Pham et al., 2011
S. stipitis NCYC1540	650	280;300	140;500	325;325	ND	Pham et al., 2011
*L. thermotolerance**	683	77; 285; 311	88; 90; 205; 300	8; 332; 343	55; 628	*KX615896*
*L. thermotolerance**	574	4; 10;285; 296	17;88;190; 300	8;270;317	-	*MZ207960.1*
*L. thermotolerance**	638	7;10;210;285	20;88;166;300	8;273;283	-	*MZ207958*
ST8.1	400	190;210	400	200;200	400	
ST10	400	190;210	400	200;200	400	
ST21.1	400	190;210	400	200;200	400	
ST21.2	400	190;210	400	200;200	400	
*M. reukaufii**	400	93;189	400	122;160	400	MW710649.1
*M. reukaufii**	400	118;151	400	89;180	400	MW710302.1
ST53	500	100;175	100;350	200;300	150;250	
P. membraniefaciens CBS 5516	479	64;69; 83;101;162	5;12;48;88; 326	205;275	ND	Pham și colab., 2011
P. membraniefaciens CECT1115	500	75;90; 110;175	50;90;330	200;275	ND	Pham și colab., 2011
P. membraniefaciens CECT10037	500	75;110;260	50;90;330	200;275	ND	Pham și colab., 2011
P. membraniefaciens CBS107	475	80;90; 110;160	50;80;320	200;275	ND	Pham și colab., 2011
P. membraniefaciens VTTV056624	500	60;80; 90;110;160	50;100;350	220;280	ND	Pham și colab., 2011
ST1	450	60;180;190	60;400	230;235	450	
C. vanderwaltii CECT11169	480	100;150;225	480	240;240	ND	Esteve-Zarzoso și colab., 1999

C. vanderwaltii CBS5524*	389	13;56; 119;201	389	161; 228	389	EU445588
*S. bombi**	473	36;56;69;104;159	44;380	8;107;109;200	40;384	MW473495.1
*S. bombi**	436	36;56;76;104;170	55;387	8;109;118;207	47;395	MH 595287.1
ST19	415	415	415	165;220	415	
P.(K) ohmeri CBS 5367	420	420	420	175;210	ND	Villa-Carvajal și colab., 2006
P.(K.) ohmeri GDB-JPCM	430	ND	ND	ND	430	Basílio și colab., 2008
*P.(K.) ohmeri**	383	383	383	15;142; 218	383	JN183446.1
*P. (K) ohmeri**	432	432	432	15;201; 208	419	EF190229.1
*P. (K.) ohmeri**	419	419	419	15;186; 210	419	KY792622.1

For strains marked * the nucleotide sequences of the amplicons were downloaded from the NCBI database and the restriction fragments were determined using the RestrictionMapper tool available online.
